# Surface Graphitized Mesoporous Carbon Surpasses the Conductivity–Porosity Trade‐Off

**DOI:** 10.1002/advs.202519661

**Published:** 2026-01-04

**Authors:** Juntian Fan, Yating Yuan, Tao Wang, Huimin Luo, Fan Wang, Qingju Wang, Shannon M. Mahurin, Bishnu P. Thapaliya, Lilin He, Jue Liu, Nikolaos Samartzis, Zhenzhen Yang, Sheng Dai

**Affiliations:** ^1^ Chemical Sciences Division Oak Ridge National Laboratory Oak Ridge Tennessee USA; ^2^ Manufacturing Science Division Oak Ridge National Laboratory Oak Ridge Tennessee USA; ^3^ Department of Chemistry Institute For Advanced Materials and Manufacturing University of Tennessee Knoxville Tennessee USA; ^4^ Neutron Scattering Division Oak Ridge National Laboratory Oak Ridge Tennessee USA

**Keywords:** electrical conductivity, electrochemical activation, mesoporous carbon, molten salts, porosity engineering

## Abstract

In carbon engineering, a longstanding trade‐off persists: chemical activation increases surface area but sacrifices conductivity, whereas graphitization enhances conductivity at the expense of porosity. In 2017, we introduced an electrochemical graphitization strategy using cathodic polarization in CaCl_2_‐NaCl molten salts to convert hard carbon into graphite. Here, we reveal that this graphitization process initiates at the surface and propagates inward, enabling the transformation of mesoporous hard carbon into surface‐graphitized mesoporous carbon. Meanwhile, this phenomenon is an electrochemical activation process: short‐term graphitization rearranges carbon atoms to increase surface area from 397 to 867 m^2^/g, without significant mass loss. Unlike chemical activation, which achieves similar surface area gains at the cost of >50% yield loss, our method maintains nearly 100% carbon yield while preserving mesoporosity. The resulting material delivers a 17‐fold increase in electrical conductivity (26–450 S/cm). This scalable, energy‐efficient approach resolves the long‐standing graphitization–porosity dilemma, producing carbons with both high conductivity and large accessible surface area.

## Graphical abstract

Electrochemical cathodic polarization in molten CaCl_2_‐NaCl converts mesoporous carbon (MC) into surface‐graphitized mesoporous graphite, simultaneously increasing surface area (397–867 m^2^/g) and electrical conductivity (26–450 S/cm) without mass loss. This method provides a scalable, energy‐efficient solution to the long‐standing graphitization–porosity dilemma in carbon materials.

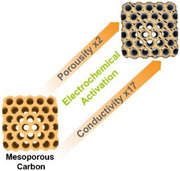

## 1 Introduction

Graphite, with its well‐ordered crystalline structure, offers higher electrical conductivity and improved resistance to electrochemical corrosion compared to amorphous carbon [1]. However, the lack of intrinsic porosity limits graphite's ability to support rapid ion transport, restricting its versatility in advanced technologies, such as high‐energy supercapacitor and high‐power sodium‐metal batteries [2, 3, 4]. Consequently, the development of porous graphite has emerged as a critical research priority [5]. To this end, the self‐assembly of nanographene and graphitization of porous carbon materials have been proposed as two promising routes. The self‐assembly of expensive nanographene often results in the formation of porous amorphous carbon, failing to achieve a well‐defined porous graphite structure [6]. Consequently, increasing attention has been directed toward the graphitization of porous carbon materials as a more feasible alternative. Among them, mesoporous carbon (MC) has attracted significant interest in materials science for its uniform and tunable pore sizes (2–50 nm), promoting the efficient transport of electrolyte ions [7, 8, 9, 10, 11], and it therefore constitutes a standout material as porous graphite precursor. However, as a typical hard carbon, mesoporous carbon cannot be graphitized by the Acheson method even at >3000°C. State‐of‐the‐art catalytic graphitization utilizing transition metals, also faces distinct challenges such as pore collapse and residual metal impurities, due to the non‐uniform distribution of metal catalysts [1, 12, 13, 14, 15]. Therefore, the terms “graphitization” and “porosity” have long been regarded as inherently contradictory. In fact, compared to the bulk graphitization procedures, controllable surface graphitization toward porous graphite—achieved by forming ultrathin layers of graphite on the surface of amorphous porous carbon and the walls of the porous channels—offers a solution to this dilemma by preserving the delicate porous framework while simultaneously transforming the rough amorphous surface into a robust, well‐defined graphitic structure. Moreover, it is noteworthy that porous graphite obtained through surface graphitization shows great potential for electrochemical applications, as detrimental reactions primarily occur at the surface, where the formation of robust and stable graphitic layers effectively mitigates adverse processes such as corrosion, thereby enhancing durability and electrochemical performance [1, 16, 17, 18, 19]. However, current surface graphitization methods such as mechanochemical processes involving the ball‐milling of porous carbon with graphite often fail to achieve uniform graphite coating and usually lead to inferior integrity of the porous structure [20]. Meanwhile, chemical vapor deposition (CVD), via the decomposition of carbon‐containing gases (e.g., methane or acetylene) at high temperatures could deposit uniform graphitic layer on the substrate but typically lead to diminished porosity [21]. Therefore, it is highly challenging yet critical to develop advanced methodologies to concurrently achieve surface graphitization and preservation or even further activation of porosity.

**FIGURE 1:**
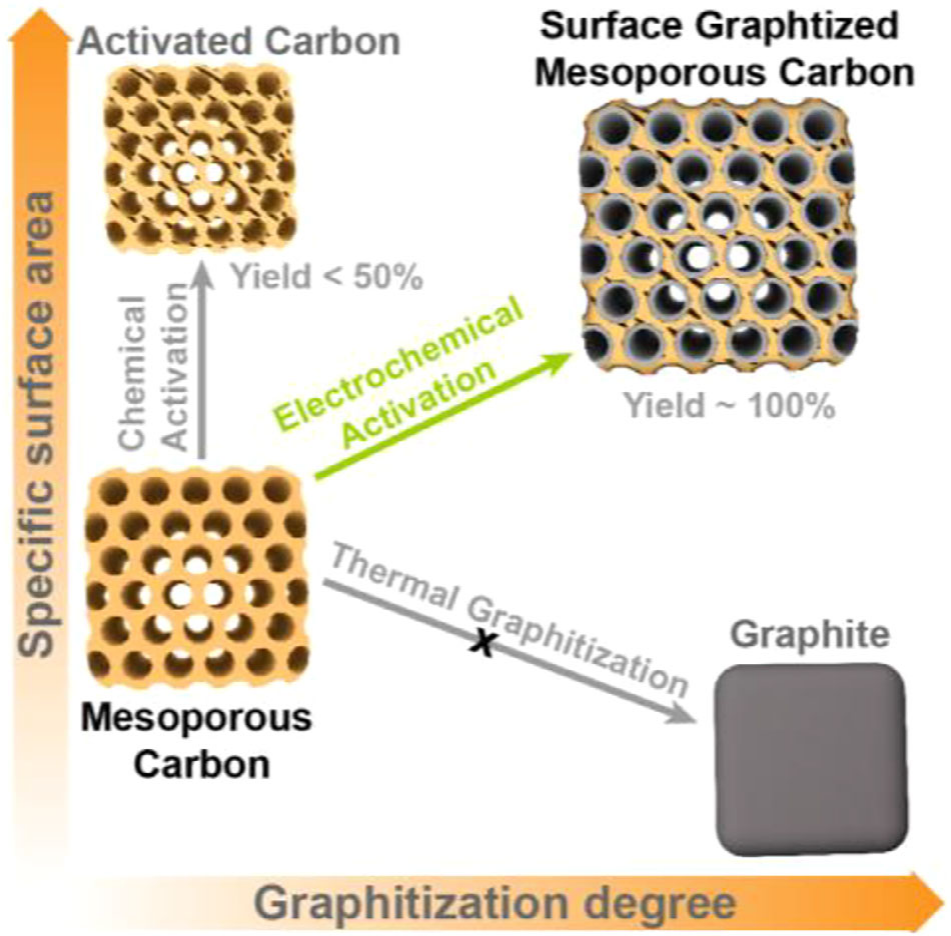
Comparison of different procedures being deployed in porosity engineering and graphitization of carbon materials.

In 2017, we introduced an electrochemical cathodic polarization method in molten salts (CaCl_2_‐NaCl) to convert hard carbon into graphite []. Herein, we reveal that this graphitization process initiates at the surface and propagates inward, enabling a uniform and controllable surface graphitization to synthesize highly conductive mesoporous carbon (Figure 1). Unlike chemical activation, which increases surface area but incurs >50% mass loss, this method retains ∼100% yield, increases surface area from 397 to 867 m^2^ g^−1^, and preserves uniform mesopores. The flux media and applied electric field enables graphitization at a much lower temperature (around 840°C) than conventional thermal methods and obviates the need for external catalysts, offering a more refined approach to enhance electrochemical performance. A short term (within 15 min) electrochemical treatment of mesoporous carbon (MC) yielded mesoporous graphite, as evidenced by a notably increased I_G_/I_D_ ratio (from 0.29 to 2.5) in Raman spectra. Neutron total scattering analysis reveals that molten salt electrochemical treatment enhances the structural coherence and graphitization degree of obtained mesoporous graphite, with improved long‐range ordering and the emergence of inter‐layer correlations. Simultaneously, the electrical conductivity of the obtained mesoporous graphite significantly increased to 450 S/cm after 15 min of treatment, 17 times the conductivity of MC, highlighting the effectiveness of the graphitization process. The unique features of the as‐afforded porous graphite open horizons for electrochemical supercapacitors, leading to increased supercapacitance and improved rate performance. The significance of tailored pore architectures in boosting electrochemical performance has also been demonstrated by He et al., who synthesized egg‐box‐like carbons with a high specific surface area and excellent areal capacitance in all‐solid‐state supercapacitors [22]. Consequently, the electrochemical cathodic polarization process developed herein provides a facile solution to the graphitization‐porosity engineering dilemma existed in the traditional carbon modification approaches and could further facilitate the application of carbon materials in energy storage with enhanced long‐term robustness.

## 2 Results and Discussion

**FIGURE 2:**
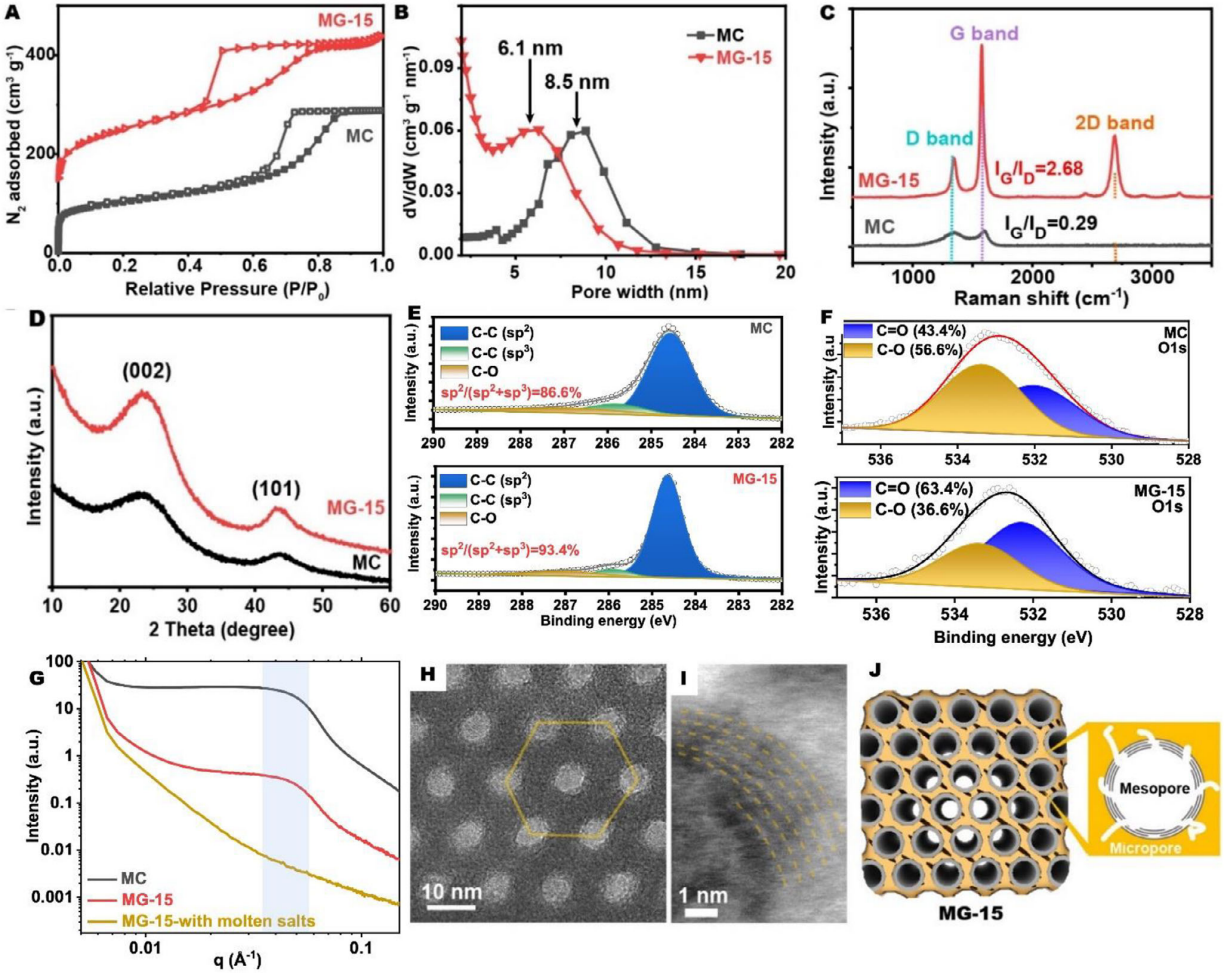
Characterizations of pristine MC and MG‐15: (A) N_2_ isotherms collected at 77 K. (B) BJH mesopore size distribution curves. (C) Raman spectra. (D) XRD patterns. (E) Deconvoluted C1s of MC and MG‐15. (F) O1s XPS of pristine MC and MG‐15. (G) SAXS spectra of MC and MG‐15 before and after removing the molten salts. (H) HR‐TEM image of MC showing hexagonal mesopores. (I) HAADF‐STEM image of MG‐15 showing in‐pore graphite structure. (J) Schematic of the pore structure changes after electrochemical graphitization.

MC with hexagonal mesoporous structure was synthesized by carbonization of a phloroglucinol–formaldehyde‐based precursor, using amphiphilic molecules (block copolymer F127) as structure‐directing agents [23]. During the electrochemical activation process, MC was immersed in molten NaCl‐CaCl_2_ at 840°C and subjected to a constant cathodic polarization of −2.6 V for specific durations to control graphitization degree and pore structure. The graphitized mesoporous carbon was denoted as MG‐T, where T represents the reaction time in minutes. As shown in Figure 2, a short cathodic polarization time of 15 min radically improves both graphitization degree and pore structure. The N_2_ isotherms of MC and MG‐15 exhibited typical Type IV profiles with a hysteresis loop (Figure 2A), indicating the preservation of mesoporous characteristics during electrochemical treatment. The Barrett‐Joyner‐Halenda (BJH) pore size distribution indicates a decreasing mesopore size (Figure 2B), from 8.5 nm (MC) to 6.1 nm (MG‐15). The Brunauer‐Emmett‐Teller (BET) specific surface area doubled, from 397 to 867 m^2^ g^−1^, while the micropore specific surface area tripled, from 139 to 409 m^2^ g^−1^. This microporosity enhancement is attributed to the reconstruction of amorphous structure to crystalline domains during cathodic polarization [24]. It should be noted that, unlike chemical activation—which increases surface area at the cost of significant carbon loss and thus low yield (typically below 50%)—our electrochemical activation enhances porosity while retaining nearly 100% of the carbon content during the reconstruction process. Notably, −2.6 V provides an optimal balance between effective graphitization and high carbon retention. Control experiments show that lowering the polarization voltage to −2.5 V does not induce graphitization even after 2 h of treatment (Figure S1), whereas increasing the voltage to −2.7 V reduces the product yield from ∼100% to ∼70%.

The graphitization of carbon network during electrochemical activation was characterized with Raman spectroscopy and powder X‐ray diffractograms (PXRD, Figure 2C,D). The Raman spectra of MC correspond to amorphous carbon, as the graphitic (G) peak from the E_2_g vibrational mode of sp^2^‐hybridized carbon atoms and the defect (D) peak from A_1_g symmetry breathing mode are broad and overlapping. The G peak to D peak intensity ratio (I_G_/I_D_) is 0.29, indicating a high defect density in the MC precursor [25]. In MG‐15, the following changes are observed: (i) the defect‐induced D peak is diminished, with I_G_/I_D_ increasing from 0.29 to 2.5 (see fitting in Figure S2); (ii) the G peak width was significantly reduced, indicating improved crystallinity; (iii) a prominent 2D peak emerges, supporting the formation of a graphitic structure. The single‐Lorentzian shape of the 2D peak, along with the absence of the M band at ∼1750 cm^−1^ and the presence of the iTALO^+^ (∼1880 cm^−1^) and iTOLA/LOLA (∼2000–2050 cm^−1^) modes (Figure S3), support the turbostratic stacking of graphitic layers [26]. The increased graphitization degree was also validated by X‐ray photoemission spectroscopy (XPS). Specifically, the C1s peaks of MC and MG‐15 were deconvoluted into C‐C (sp^2^) located at 284.5 eV, C‐C (sp^3^) at 285.8 eV, and C‐O at 286.9 eV (Figure 2E). Although the types of carbon species remain the same before and after treatment, their relative content changes significantly after the electrochemical treatment. For instance, the surface oxygen content of MC is 2.7 at%, while that of MG‐15 drops to 0.7% [17]. In addition, the sp^2^ C content rises from 86.6% in MC to 93.4% in MG‐15, confirming an increased graphitization degree after electrochemical treatment [27]. Moreover, the O1s spectra of MC and MG‐15 reveal that during electrochemical treatment, C‐O groups are more readily eliminated compared to C = O groups (Figure 2F). While Raman and XPS spectra showcase the formation of a crystalline graphitic structure, the PXRD patterns of MC and MG‐15 indicate the bulk amorphous structure (Figure 2D). This apparent contradiction can be reconciled by the fact that Raman (for black carbon) and XPS, being a surface‐sensitive technique, confirms that the graphitization of MC achieved here is predominantly localized on the surface. To gain a deeper insight into the porosity changes during the graphitization and porosity engineering processes, small‐angle X‐ray scattering (SAXS) techniques, capable of probing ordered mesopore structures, were conducted. As illustrated in Figure 2G, the peak observed at 0.05 Å^−1^ corresponds to the (100) reflection of hexagonal mesopore structures [28]. The hexagonal mesoporous structure was validated by the high‐angle annular dark‐field scanning transmission electron microscopy (HAADF‐STEM) image (Figure 2H). The SAXS pattern of MG‐15 prior to the removal of the molten salts revealed the complete attenuation of the (110) peak, indicating that the mesopores were occupied by the molten salts during electrochemical graphitization (Figure 2G). The (110) peak appears again in the SAXS pattern of MG‐15 after the removal of salts confirming the well preserved ordered mesopores after electrochemical activation, which is consistent with the N_2_ isotherm results presented in Figure 2A,B. Moreover, the formation of graphitic structure on mesopore surface was directly observed by STEM, which shows that the MC with an amorphous structure without lattice fringes (Figure 2H). As a contrast, the HAADF‐STEM image of MG‐15 reveals a set of lattice fringes with a spacing of 0.36 nm along the mesopore wall, corresponding to the (002) plane of graphite with sixfold symmetry (Figure 2I) [18]. The results revealed well‐preserved mesopores, with graphite stacks consisting of several well aligned graphene sheets observed on the pore walls of the mesoporous structure. This interlayer spacing is considerably larger than that of highly‐oriented pyrolytic graphite (0.335 nm) and is typical for turbostratic graphite, indicating the electronic decoupling of adjacent graphene layers induced by rotational stacking faults [29]. As schematically depicted in Figure 2J, the growth of graphitic layers around the pore walls of MC could explain the decreased mesopore size after the electrochemical treatment. Compiling the above‐mentioned insights, the graphitization of amorphous MC occurs at its interface with the molten salt, resulting in the formation of thin turbostratic graphite stacks on mesopore wall in MG‐15.

**FIGURE 3:**
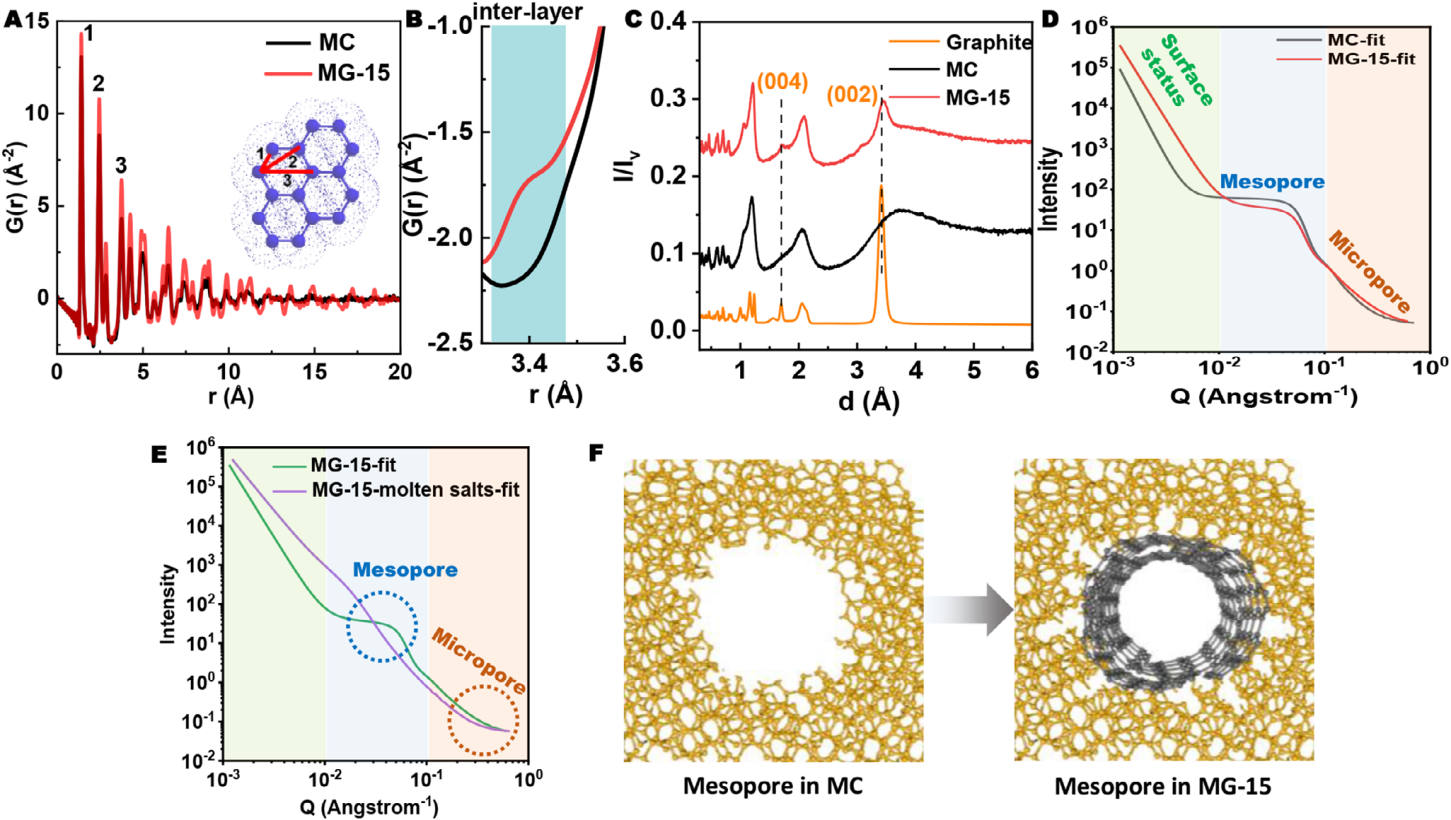
(A) Atomic pair distribution functions in the configuration of the reduced radial distribution function. (B) Enlarged view from 3.3 to 3.6 Å. (C) Neutron diffraction patterns of graphite, MC, and MG‐15. (D) SANS fitting curves of MC and MG‐15. (E) SANS fitting curves of MG‐15 before and after removing the molten salts. (F) Illustration of mesopore structure changes during electrochemical graphitization.

To further elucidate the local atomic structures of MC and MG‐15, neutron total scattering experiments were conducted. The peaks of the atomic pair distribution function G(r) represent the probability density of spatial correlations between carbon atoms. As shown in Figure 3A, MG‐15 and MC exhibit similar features in the low r‐region, with prominent peaks at approximately 1.4, 2.4, and 2.8 Å, corresponding to the characteristic 1,2‐, 1,3‐, and 1,4‐carbon‐carbon distances [30]. The intensity of the G(r) peaks of MC diminishes beyond approximately 12 Å, indicating its limited structural coherence. In contrast, the coherent length (a measure of crystallinity) of MG‐15 increases, signifying its improved long‐range ordering compared to MC (Figure 3A) [31]. The short‐range G(r) analysis highlights changes in the distribution of C‐C pairs, where peak 3, specific to six‐membered rings, reflects sp^2^ carbon, while peak 1 appears in all types of carbon, including sp^3^ carbon and defects (Figure 3A). By comparing the ratio of peak 3 to peak 1, it is evident that the proportion of sp^2^ carbon of MG‐15 is higher than that of MC (0.28 *vs* 0.21). Moreover, MG‐15 exhibits a distinct peak at 3.36 Å, which does not appear in MC and coincides precisely with the inter‐layer atomic correlations characteristic of graphite, further confirming its mesoporous graphite structure (Figure 3B) [32]. The neutron diffraction patterns were also collected to demonstrate the structural evolution of MG‐15. As shown in Figure 3C, the neutron diffraction pattern of MG‐15 exhibited the (002) and (004) peaks, which were not observed in the pattern of MC, reflecting the enhanced graphitization in MG‐15. The (002) and (004) peaks of MG‐15 shifted to the right compared to those of graphite, suggesting that the interlayer distance of the graphite layers is larger than that of commercial graphite. This is consistent with the previous Raman results and suggests that the graphite layers exhibit a turbostratic stacking arrangement. The detailed structure evolution on carbon interface in MG‐15 was further characterized by small‐angle neutron scattering (SANS), which is more sensitive to light elements than X‐ray [33]. The curves of the pristine MC and MG‐15 were collected at the general‐purpose SANS (GP‐SANS) diffractometer covering a q‐range of 0.004< q < 0.5 Å^−1^. The scattering profiles of these samples follow a combined model with power law decay in the low q regime, an interacting polydisperse sphere in middle q regime, and a correlation length model at high q regime, as the fitted results were summarized in Table S1. The surface property variation upon electrochemical treatment was compared focusing on the low Q range. The as‐derived porod exponent of the pristine MC from fitting was 5.04 (Figure 3D; Table S1), indicating a fuzzy surface of the granule particles [34]. After the short‐term graphitization treatment, the porod exponent value around 4.20 (less steeper) was obtained for MG‐15, revealing the formation of a smoother surface. In the middle Q region, both MC and MG‐15 exhibited pronounced shoulder peaks, corresponding to the presence of permanent mesoporous channels. The mean radius from the fitting results revealed a slight decrease in terms of the mesopore size after electrochemical treatment, consistent with the N_2_ isotherm characterizations (Figure 2B), which was also elucidated by the diminished neutron scattering intensity. It should be noted that neutron scattering provided pore size information reflected by the variation of nucleus distribution instead of the gas adsorption behavior (N_2_ isotherms), leading to minor differences in the as‐derived pore size results from these two methods [35]. Comparatively, in the high Q region reflecting the variation of micropore property, increased neutron scattering intensity of the MG‐15 was observed, surpassing that of the pristine MC, supporting the creation of more micropores during the electrochemical treatment procedure. Furthermore, distribution of the molten salts in the MG‐15 was probed by comparing the SANS profiles before and after the molten salts was removed (Figure 3E). The neutron scattering signal in the middle and high Q range reveals the filling of mesoporous and microporous channels by molten salts, which is consistent with the SAXS results (Figure 2G). As the graphitization process occurred at the interface of carbon‐molten salts phase, this provided strong support on the formation of thin layer graphite on the surface of the porous channels. Compared to the amorphous structure, the well‐aligned graphitic structure leads to reduced surface roughness within the mesopores. Figure 3F illustrates the distinct mesopore wall structures of MC and MG‐15, highlighting the improved long‐range ordering and reduced surface roughness in MG‐15.

**FIGURE 4:**
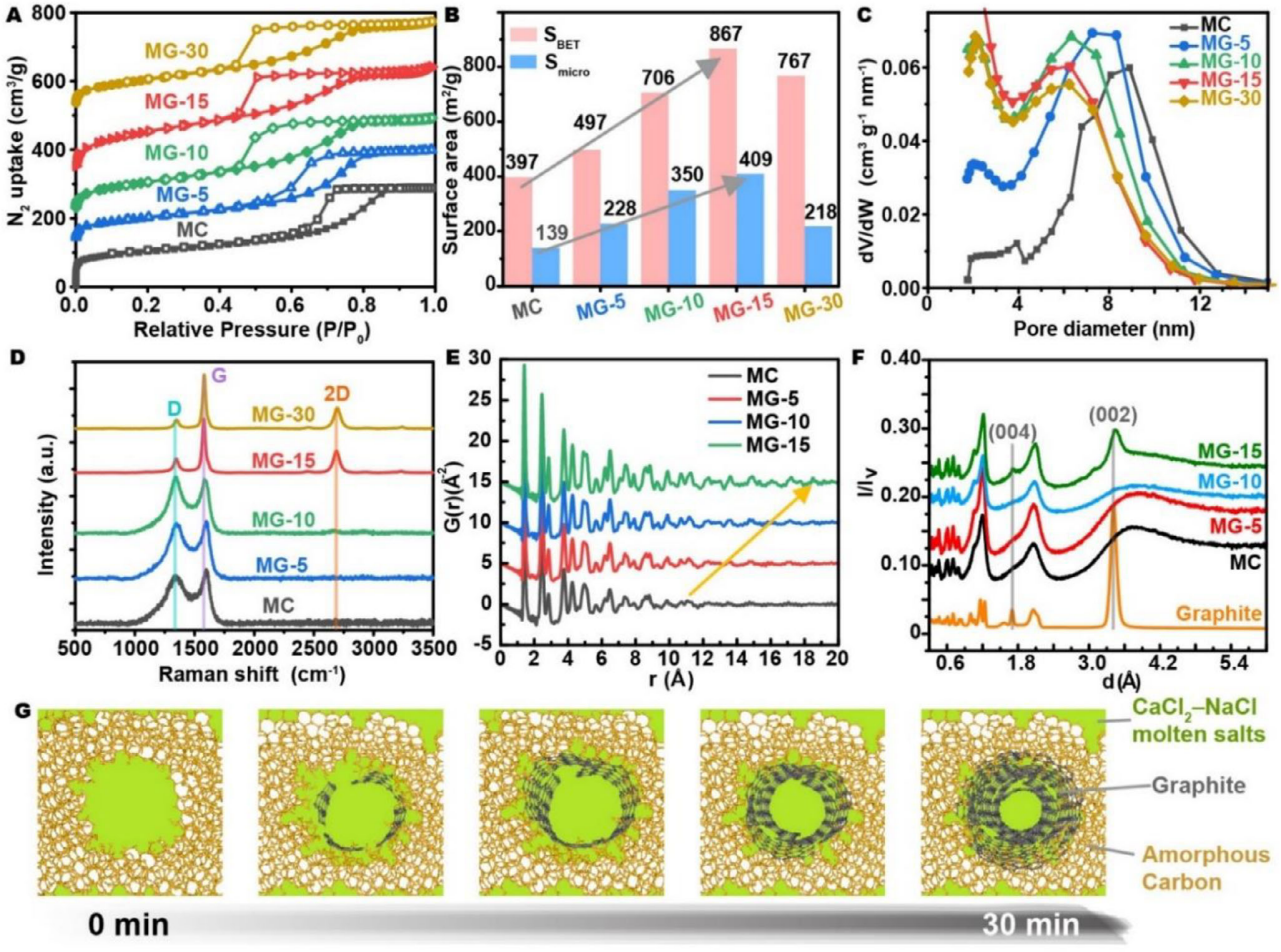
Characterizations of pristine MC and MG‐T treated by electrochemical cathodic polarization for different time scale: (A) N_2_ isotherms collected at 77 K (values are offset along the y‐axis by 58 cm^3^ g^−1^ (blue), 100 cm^3^ g^−1^ (green), 202 cm^3^ g^−1^ (red), 427 cm^3^ g^−1^ (yellow). (B) BET and microporous surface areas. (C) BJH mesopore size distribution curves. (D) Raman spectra. (E) Atomic pair distribution functions in the configuration of the reduced radial distribution function. (F) Neutron diffraction patterns. (G) Mesopore structure evolved over time during the electrochemical activation process.

Building on the well‐defined structural features of MG‐15, additional MG‐T were prepared with shorter (5 and 10 min) and longer (30 min) treatment durations to systematically investigate the time‐dependent evolution of structural features. The Type‐IV N_2_ physisorption isotherms of all mesoporous samples are presented in Figure 4A. A short treatment of only 5 min increased the BET specific surface area from 397 to 497 m^2^ g^−1^, elevating the micropore surface area from 139 to 228 m^2^ g^−1^. Increasing the treatment time raised the surface area up to 867 m^2^ g^−1^ (MG‐15), with a corresponding micropore surface area of 409 m^2^ g^−1^, before subsequently decreasing(Figure 4B; Table S2). Therefore, short‐term treatment did not compromise the integrity of the internal pore structure upon graphitization; rather, it appears to progressively augment microporosity and enhance the overall surface area. However, prolonged graphitization ultimately degrades the internal pore structure, reducing surface area (Figure 4B). Notably, the BJH‐derived mesopore size decreased with increasing treatment time (9 nm for MC, 8 nm for MG‐5, and 6 nm for MG‐10/15/30) (Figure 4C), which may be attributed to the introduction of non‐porous graphite layers during the graphitization process. Considering carbon quality evolution, the PXRD of Figure S4 proves the preservation of an amorphous bulk structure throughout a treatment time of 30 min. On the contrary, Raman spectroscopy revealed the gradual surface graphitization during cathodic polarization. In specific, increasing the treatment time reduces the D peak monotonously, with the I_G_/I_D_ area ratio elevating from 0.29 (MC) to 0.32 (MG‐5), 0.34 (MG‐10), 2.5 (MG‐15), and 5.0 (MG‐30), while the G peak sharpens, suggesting an increasing domain size. Also, a symmetric 2D peak emerges in MG‐15/30, indicative of a non‐Bernal stacked graphitic structure (Figure 4D). According to the C1s XPS peaks, the sp^2^ carbon content of MC's surface is monotonously enriched from 86.6% (Figure 2E) to 88.6% in MG‐5 (Figure S5), 93.4% in MG‐15 (Figure 2E), and 94% in MG‐30 (Figure S6). To unveil the local atomic structures of MC and MG‐T, neutron total scattering experiments were conducted. The peaks of the atomic pair distribution function G(r) represent the probability density of spatial correlations between carbon atoms. As shown in Figure 4E, MG‐T and MC exhibit similar features in the low r‐region, with prominent peaks from the characteristic 1,2‐, 1,3‐, and 1,4‐carbon‐carbon distances [30]. The coherent length (a measure of crystallinity) increases with the duration of molten salt electrochemical treatment, signifying improved long‐range ordering in MG‐T compared to MC (Figure 4E) [31]. The neutron diffraction patterns were also collected to demonstrate the structural evolution of MG‐T. As shown in Figure 4F, the (002) and (004) peaks of MG‐T gradually emerged with increasing graphitization time, reflecting the enhanced graphitization. The electrochemical activation mechanism, informed by the time‐dependent activation data, is illustrated in Figure 4G and Figure S7. Applying a negative voltage during heating promotes the graphitization of amorphous carbon by injecting electrons, which raise the Fermi level and weaken disordered C–C bonds. This electron‐driven mechanism lowers the activation energy required for structural reorganization, enabling a more efficient transformation into a thermodynamically stable graphite structure. The reorganization initiates at the interface between carbon and molten salts, where graphene domains begin to form on the mesopore surfaces. As activation time increases, these domains grow, merge, and stack into graphite layers along the mesopore walls, resulting in reduced pore size and smoother surfaces. Simultaneously, the conversion of amorphous carbon to denser graphite induces the formation of micropores at the interface between the newly formed graphite layers and the remaining amorphous matrix. With prolonged activation, the graphitic structure progressively extends into the carbon backbone, ultimately leading to a reduction in overall surface area. Moreover, to further elucidate the surface‐to‐interior graphitization, TEM was conducted and is shown in Figure S8. The pristine mesoporous carbon shows no observable lattice fringes, whereas 15 min of treatment results in approximately six graphitic layers at the pore walls, 30 min yields ∼8 layers, and 1 h produces over 13 layers, providing direct evidence of the time‐dependent deepening of graphitic ordering.

**FIGURE 5:**
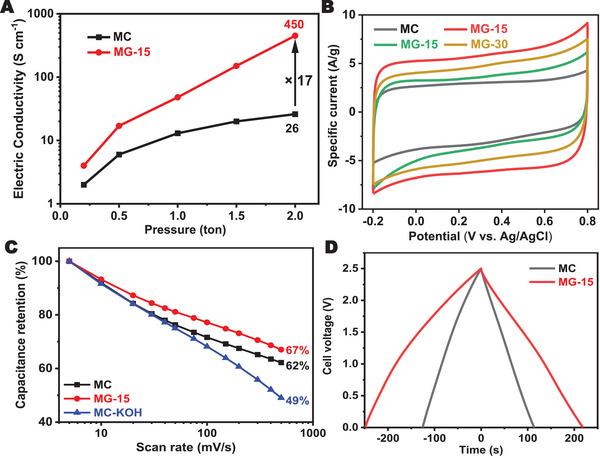
(A) Electrical conductivity of MC and MG‐15 under elevated pressure. Electrochemical performance of MC and MG‐T in 1 M H_2_SO_4_: (B) CV curves at the scan rate of 50 mV s^−1^. (C) Rate performance at scan rates from 5 to 500 mV s^−1^. Electrochemical performance of symmetric supercapacitor of MC and MG‐15 with 1 M NEt_4_BF_4_ as electrolyte: (D) Galvanostatic charge‐discharge curves at 0.2 A g^−1^.

After demonstrating the successful synthesis of mesoporous graphite via electrochemical polarization, its electrical conductivity was tested, as graphitization is known to enhance conductivity by increasing structural order and promoting sp^2^‐hybridized carbon domains, which facilitate efficient electron transport. Herein, the MG‐15 was selected to test the electrical conductivity due to its largest surface area. As shown in Figure 5A, the electric conductivity of MG‐15 reaches 450 S/cm, 17 times higher than that of MC. Given that the electrical conductivity of particulate materials may depend on structural orientation, the microstructure of the compressed MG‐15 was examined and found to be randomly packed without detectable alignment (Figure S9), indicating that the measured conductivity reflects isotropic bulk behavior. As previously noted, the electrochemical treatment of MC leads to a notable increase in BET surface area and pore volume, along with an enhancement in electronic conductivity due to graphitization, all of which are paramount for supercapacitor applications. In light of this, MG‐T were systematically evaluated in comparison to the original MC using a traditional three‐electrode setup in a 1 M H_2_SO_4_ electrolyte for supercapacitive performance. As shown in Figure 5B and Figures S10–S13, the cyclic voltammetry (CV) curves of both pristine MC and the MG‐T display quasi‐rectangular shapes, indicative of the predominance of electric double‐layer capacitance (EDLC). It should be noted that the deviations from an ideal rectangular shape of CV curves suggest the presence of pseudocapacitance, likely originating from oxygen‐containing functional groups (Figures S10–S13), which are derived from the polymerization of phloroglucinol and formaldehyde. In addition, although the oxygen content is reduced during the molten salt cathodic process for MG‐T, residual oxygen still contributes to the observed pseudocapacitive effects [36]. To further differentiate the contributions of capacitive and pseudocapacitive processes, kinetic analysis was carried out based on CV data collected at different scan rates (Figures S10–S13). Typically, the relationship between the current density (i) and the scan rate (ν) follows the equation i = a*ν*^b^*, where b values of 1, 0.5, and between 0.5 and 1 correspond to ideal capacitive behavior, diffusion‐controlled kinetics, and a mix of diffusion and capacitive behavior, respectively [37]. By plotting the logarithm of current density against the logarithm of the scan rate (log *i* = *a* + *b* logν), the b values for MC, MG‐10, MG‐15 and MG‐30 were calculated to be 0.904, 0.925, 0.929 and 0.935, respectively (Figure S14). The increase in the *b* value indicates a higher contribution from electric double‐layer capacitance (EDLC), which aligns with the improved electronic conductivity by the graphitization process. Furthermore, the specific capacitance of pristine MC and MG‐T was evaluated through the integration of their cyclic voltammetry (CV) curves. As shown in Figure S15, the capacitance is highest for MG‐15 at all scan rates, followed by MG‐30, MG‐10, and MC, demonstrating that MG‐15 provides the optimal balance between conductivity and accessible surface area and achieves the best overall performance for supercapacitor applications. More specifically, pristine MC exhibited a specific capacitance of 78.4 F g^−1^ at a scan rate of 5 mV s^−1^, which further decreased to 48.8 F g^−1^ at 500 mV s^−1^, with a capacitance retention of only 62%, indicating its limited electrochemical performance. In contrast, MG‐15 achieved a superior specific capacitance of 148.3 F g^−1^ at 5 mV s^−1^, and importantly, retained an impressive 99.4 F g^−1^ even at the high scan rate of 500 mV s^−1^, which corresponds to an enhanced capacitance retention of 67% (Figure S15; Figure 5C). To further demonstrate the advantage of mesoporous graphite achieved herein, a KOH‐activated MC with a comparable surface area (691 m^2^ g^−1^) was synthesized for comparison (Figure S16), which exhibited a markedly lower capacitance retention of 49%, significantly inferior to the 67% achieved by MG‐15 (Figure 5C). To further examine long‐term structural stability, MG‐15 was subjected to a voltage‐holding test in which a constant potential of 0.4 V vs. Hg/Hg_2_SO_4_ was applied with intermittent galvanostatic charge–discharge cycles [38]. The capacitance retention remained almost unchanged after 400 h, confirming that the graphitized mesoporous framework maintains its integrity during extended electrochemical operation (Figure S17). Subsequently, MC and MG‐15 were further evaluated in symmetric supercapacitors using 1 M tetraethylammonium tetrafluoroborate (NEt_4_BF_4_) in anhydrous acetonitrile (ACN) as the electrolyte, enabling a wide operating voltage window of 2.5 V. As shown in Figure S18, MG‐15 deviated considerably from the EDLC‐type behavior, with CV features similar to those of pseudocapacitance. This is attributed to the nanoconfined formation of the electric double layer [39], as the micropores are too small to allow the diffusion of solvated electrolyte ions, necessitating their partial desolvation [40]. Apart from the CVs, this effect is also evident from the enlarged high‐frequency semicircle of the Nyquist plot of MG‐15 (Figure S19), associated with the charge transfer resistance [41]. Nyquist plots also support the increased electronic conductivity of MG‐15, as the equivalent series resistance drops from 2.1 Ohm (MC) to 1.1 Ohm (MG‐15). The device capacitance was extracted from the galvanostatic charge/discharge (GCD) curves of MC and MG‐15 (Figure S20). At the specific current of 0.05 A g^−1^, MC exhibited a specific capacitance of 19.9 F g^−1^, which nearly doubled to 37.2 F g^−1^ for MG‐15 (Figure S21), agreeing to the surface area enhancement. Furthermore, the assembled symmetric supercapacitor exhibits a competitive energy density of 8.1 Wh kg^−1^ at a corresponding power density of 31 W kg^−1^, and 6.5 Wh kg^−1^ at 625 W kg^−1^, significantly outperforming MC (4.3 and 3.6 Wh kg^−1^ at the corresponding power densities, respectively) (Figure S22). Overall, the enhanced supercapacitive performance of MG‐15 showcases the significant merits of electrochemical graphitization for boosting the electrochemical energy storage capabilities of other porous carbons.

## 3 Conclusion

Surface graphitized mesoporous carbon without any metal impurities was successfully synthesized through an electrochemical activation method in molten salts, achieved by simultaneously incorporating surface graphitization and porosity engineering. This electrochemical activation approach successfully overcomes the inherent limitations of conventional methods, where graphitization typically leads to porosity loss, while activation reduces conductivity. MG‐15 exhibited a high surface area of 867 m^2^ g^−1^, more than double that of its precursor (397 m^2^ g^−1^), with abundant micropores generated and well preserved mesopores. The reconstruction mechanism of electrochemical activation overcomes the low carbon yield associated with the etching process in conventional chemical activation, enabling a carbon yield close to 100%. Nanosized turbostratic graphite structure on mesopore wall, was evidenced by increased intensity of G and 2D peaks in Raman spectroscopy, a higher sp^2^ ratio in XPS analysis, enhanced coherent length and distinct peak coinciding with the inter‐layer atomic correlations characteristic of graphite in neutron atomic pair distribution function, and graphite wrinkles in microscopy images. The surface‐graphitized mesoporous carbon, alongside with more abundant pore structure, exhibited 17 times higher electric conductivity and notably improved rate performance in supercapacitor applications.

## Conflicts of Interest

The authors declare no conflicts of interest.

## Supporting information




**Supporting File**: advs73567‐sup‐0001‐SuppMat.docx.

## Data Availability

The data that support the findings of this study are available from the corresponding author upon reasonable request.
