# Supplementary material for: Surface Graphitized Mesoporous Carbon Surpasses the Conductivity–Porosity Trade‐Off
Source: Adv Sci (Weinh). 2026 Jan 4;13(14):e19661. doi: 10.1002/advs.202519661 (PMC12970259; doi:10.1002/advs.202519661)
Supplement: Supplementary file 1 — Supporting File: advs73567‐sup‐0001‐SuppMat.docx. [file ADVS-13-e19661-s001.docx]

Supporting Information

**Surface Graphitized Mesoporous Carbon Surpasses the Conductivity–Porosity Trade-Off**

Juntian Fan,^a^ Yating Yuan,^a^ Tao Wang,^a*^ Huimin Luo,^b^ Fan Wang,^c^ Qingju Wang,^c^ Shannon M Mahurin,^a^ Bishnu P. Thapaliya,^a^ Lilin He,^d^ Jue Liu,^d^ Nikolaos Samartzis,^c^ Zhenzhen Yang,^a^ Sheng Dai ^a, c*^

^a^ Chemical Sciences Division, Oak Ridge National Laboratory, Oak Ridge, TN 37831, USA

^b^ Manufacturing Science Division, Oak Ridge National Laboratory, Oak Ridge, TN, 37831, USA

^c^ Department of Chemistry, Institute for Advanced Materials and Manufacturing, University of Tennessee, Knoxville, TN 37996, USA

^d^ Neutron Scattering Division, Oak Ridge National Laboratory, Oak Ridge, TN 37831, USA

Email: wangt@ornl.gov; dais@ornl.gov

Table of contents

[**Experimental Procedures** 3](#_Toc215402811)

[**1. Materials** 3](#_Toc215402812)

[**2. Synthesis of Mesoporous carbon (MC)** 3](#_Toc215402813)

[**3. Chemical activation of MC by KOH** 4](#_Toc215402814)

[**4. Electrochemical activation process** 4](#_Toc215402815)

[**5. Characterizations** 4](#_Toc215402816)

[**6. Electrochemical Measurements** 5](#_Toc215402817)

[**Supplementary figures** 6](#_Toc215402818)

[**Figure S2.** Representative fitting of the 900-1900 cm^-1^ region of the Raman spectra, using Lorentzian functions of MG-15. 7](#_Toc215402819)

[**Figure S3.** Raman combination bands in MG-15, testifying for the turbostratic arrangement of the graphitic layers. 8](#_Toc215402820)

[**Figure S4**. PXRDs of MC and MG-T. 8](#_Toc215402821)

[**Figure S5.** C1s XPS of MG-5. 8](#_Toc215402822)

[**Figure S6.** C1s XPS of MG-30. 9](#_Toc215402823)

[**Figure S7.** The energy diagram illustrates the mechanism by which applying a negative voltage (−V) during heating promotes the transformation of amorphous carbon into graphite. 9](#_Toc215402824)

[**Figure S8**. TEM images of pristine MC (A), MG-15(B), MG-30(C), MG-60(D). 10](#_Toc215402825)

[**Figure S10.** CV curves of MC at different scan rates. 10](#_Toc215402826)

[**Figure S11.** CV curves of MG-10 at different scan rates. 11](#_Toc215402827)

[**Figure S12.** CV curves of MG-15 at different scan rates. 11](#_Toc215402828)

[**Figure S13.** CV curves of MG-30 at different scan rates. 12](#_Toc215402829)

[**Figure S14.** The logarithm of the current versus the logarithm of the scan rate, including the fitting line based on CV curves recorded at scan rates of 5, 10, 50, 100, and 200 mV s^−1^ 12](#_Toc215402830)

[**Figure S15.** Specific capacitance calculated by the integration of CV curves using 1 M H_2_SO_4_ as electrolyte. 13](#_Toc215402831)

[**Figure S16.** Characterization of MC activated by KOH at 700 °C for 2h: (A) PXRD. (B) N_2_ isotherm at 77 K. 13](#_Toc215402832)

[**Figure S17**. Voltage holding test using three-electrode configuration at room temperature by applying a constant up-limit potential (0.4 V vs Hg/Hg_2_SO_4_) during aging for up to 400 h. Every 10 h, three GCD cycles were performed between -0.6 V and 0.4 V vs Hg/Hg_2_SO_4_ using a specific current of 5 A g^-1^. 14](#_Toc215402833)

[**Figure S18.** CV curves of MC and MG-15 at the scan rate of 10 mV s^-1^ using symmetric cells in 1 M tetraethylammonium tetrafluoroborate (NEt_4_BF_4_) in anhydrous acetonitrile (ACN) electrolyte. 14](#_Toc215402834)

[**Figure S19.** Nyquist plot comparison of MC and MG-15 15](#_Toc215402835)

[**Figure S20**. GCD curves of symmetric supercapacitors using 1M NEt_4_BF_4_ in ACN electrolyte, using (A) MC and (B) MG-15 electrode materials. 16](#_Toc215402836)

[**Figure S21.** Specific capacitance calculated from GCD curves for symmetric supercapacitors using 1M NEt_4_BF_4_ in ACN electrolyte. 16](#_Toc215402837)

[**Figure S22.** Ragone plot (energy density vs power density) for symmetric supercapacitors using 1M NEt_4_BF_4_ in ACN electrolyte. 16](#_Toc215402838)

[**Table S1** Fitting parameters of combined model with interacting polydisperse sphere and correlation length. 17](#_Toc215402839)

[**Table S2**. Summary of the Surface Area, Porosity of MC before and after electrochemical treatment 18](#_Toc215402840)

**Experimental Procedures**

**1. Materials**

Pluronic F127, phloroglucinol, formaldehyde, sodium chloride, potassium chloride, potassium hydroxide, ethanol, hydrochloric acid, were purchased from commercial sources (Fisher Scientific, Sigma-Aldrich) and were used without further purification.

**2. Synthesis of** **Mesoporous carbon (MC)**

The mesoporous carbon was synthesized based on literature.^[1]^ To be specific, mesoporous carbon particles were synthesized by carbonization of nanostructured polymeric composites, which were obtained by self-assembly of block copolymer (e.g., Pluronic F127) and phenolic resin (e.g., phloroglucinol-formaldehyde) under acidic conditions via the soft-template method. In a typical synthesis, a 2 L flask was charged with 26.2 g of phloroglucinol, 52.4 g of F127, and 10.0 g of aqueous HCl (37 wt%) in 1300 mL of ethanol (200 proof). The mixture was heated with stirring to reflux. To this solution, 26.0 g of formaldehyde aqueous solution (37 wt%) was added. Precipitates appeared at about 4 min after the addition of formaldehyde, indicating the formation of F127-phenolic resin polymeric composites. The reaction mixture was stirred for 2 h and then filtered. The yellow polymer particles were washed with ethanol and dried in an oven at 120 °C for 3 h. Carbonization was carried out under flowing N_2_ by heating the polymer particles to 850 °C with a heating rate of 2 °C min^-1^ and maintaining the final temperature for 2 h.

**3. Chemical activation of MC by KOH**

In a typical synthesis, MC and KOH were thoroughly mixed at a mass ratio of 1:1, followed by thermal treatment at 700 °C under a nitrogen atmosphere for 2 hours. The resulting product was then washed with deionized water and dilute hydrochloric acid to remove residual salts, yielding the final MC-KOH sample.

**4. Electrochemical activation process**

A total of 288 g of anhydrous calcium chloride (CaCl_2_) and 140 g of sodium chloride (NaCl) were loaded into an alumina crucible, which was then placed inside a sealed quartz reactor. The reactor was initially heated to 300 °C under a dynamic vacuum for 24 hours to remove moisture. Subsequently, the reactor was heated to 840 °C at a ramp rate of 2 °C per minute under an argon atmosphere (moisture and oxygen content < 1.0 ppm). Once the salt mixture had melted, pre-electrolysis of the molten CaCl_2_/NaCl was carried out to remove residual moisture and redox-active impurities. This was done using a graphite rod anode (~12.7 mm diameter, ~6 cm immersed in the molten salt bath) and a stainless-steel mesh cathode (~4 cm²) attached to a molybdenum rod (~2 mm in diameter), operated at 2.7 V for approximately 5 hours. The electrochemical activation process was performed by applying cathodic polarization at a constant voltage. Approximately 200 mg of the amorphous carbon precursor was wrapped in nickel foam and connected to a molybdenum rod to serve as the cathode. The previously mentioned graphite rod was used as the anode. A voltage of 2.6 V was applied between the cathode and anode at 840 °C for specified durations using a Gamry Reference 3000 electrochemical workstation. Following the electrochemical process, the product was removed from the reactor, cooled, washed with distilled water, and dried at 100 °C in a vacuum oven.

**5. Characterizations**

The nitrogen adsorption isotherms were measured at 77 K under a 3Flex, Micromeritics instrument. The samples were outgassed at 120 °C for 16 h before the measurements. Surface areas were calculated from the adsorption data using Brunauer-Emmett-Teller (BET) methods. The powder X-ray diffraction (PXRD) data were recorded with a PANalytical Empyrean diffractometer, operated at 45 kV and 40 mA (scanning step: 0.026° per step). The diffraction patterns were recorded in the range of 5-60°. λ = 0.1540598 nm. Raman spectroscopy was performed by a Renishaw In-Via Raman spectrum instrument with emission laser wavelength of 532 nm. X-ray photoelectron spectroscopy (XPS) measurements: XPS experiments were performed with a PHI 3056 spectrometer equipped with an Al anode source operated at 15 KV and an applied power of 350 W and a pass energy of 93.5 eV. Samples were mounted on foil since the C1s binding energy was used to calibrate the binding energy shifts of the sample (C1s = 284.8 eV). High-angle Annular Dark-field Scanning Transmission Electron Microscopy (HAADF-STEM) was conducted on an aberration-corrected Spectra 300 TEM. Small- angle X-ray scattering (SAXS) experiments were carried out using Xenocs Xeuss 3.0 equipped with a Cu anode X-ray generator. The small angle neutron scattering (SANS) data were collected at the GP-SANS instrument, HFIR. The instrument configurations were set to cover a q-range of 0.004 < q < 0.5 Å^-1^. The small angle neutron scattering (SANS) data were collected at the GP-SANS instrument, HFIR. The instrument configurations were set to cover a q-range of 0.004 < q < 0.5 Å^-1^. The scattering profiles follow a combined model with power law decay in low q regime, an interacting polydisperse sphere in middle q regime, and a correlation length model at high q regime.

$$I\left( q \right)=\frac{A}{q^{n}}+\frac{B}{1+{(q)}^{m}}+C+I_{inc}$$

where A, B are the prefactors, $n$is the exponent of the power law decay, is the correlation length describing the average size of the material inhomogeneity. *C* is the term from interacting polydisperse spheres. $I_{inc}$ is the incoherent scattering constant. The fitting parameters are listed in the tables (Table S1). Neutron diffraction and pair distribution function (PDF) analyses were performed using the NOMAD beamline at the Spallation Neutron Source (SNS), located at Oak Ridge National Laboratory (ORNL). Powdered samples of MC and MG-T (approximately 0.15–0.2 g each) were sealed in 3 mm-diameter thin-walled quartz capillaries for measurement. To enhance signal-to-noise ratios, four scans of 24 minutes each were conducted per sample and subsequently averaged. The background contribution from an empty quartz capillary was subtracted, and intensity normalization was carried out using a 6 mm vanadium standard to correct for variations in detector efficiency. For the PDF data reduction, a Q_max_ cut-off of 50 Å^−1^ was adopted for all samples during Fourier transform of the S(Q) to the reduced PDF G(r) or pair distribution function g(r).

**6. Electrochemical Measurements**

The electrochemical performance of the as-prepared mesoporous carbons and the surface-graphitized counterparts were tested on a CHI 760E electrochemical analyzer using three-electrode configuration. The three-electrode configuration was composed of a working electrode, counter electrode (Pt wire) and reference electrode (Ag/AgCl in 1M KCl). The working electrode was prepared by drop-casting homogenous ink which contains the active materials, carbon black and Nafion binder on a polished glass-carbon electrode. To be specific, 5 mg of active materials, 1 mg of carbon black as conducting agent, and 7.5 μL of Nafion solution (5 wt%) as binder were dispersed in 1 mL of isopropanol by sonication for 1 hour to form a homogeneous ink. For electrochemical capacitance test, 10 μL ink was drop-casted onto a polished glassy carbon rotating disk electrode of 0.196 cm^2^ surface, and the final electrode was obtained with a loading amount of 0.255 mg cm^−2^. The electrolyte for electrochemical capacitance test is 1 M H_2_SO_4_ as electrolyte. Cyclic voltammetry (CV) measurements were carried out at different scan rates from 5 to 500 mV s^−1^ and the specific supercapacitance from the integrated CV curves by equation (1).

$$C= \frac{\int IdU}{2mv\Delta U} (1)$$

where $\int IdU$ is the integral area of the cyclic voltammogram loop, ΔU is the sweep potential window, v is the scan rate, and m is the mass of the electrode material.

For the symmetric supercapacitors, a coin cell configuration was considered, using a 1M NEt_4_BF_4_ in dry ACN as electrolyte, glass fiber as separator. The capacitance of the symmetric supercapacitors was calculated from the galvanostatic charge-discharge (GCD) curves, using equation (2).

$$C_{spec;dev}=\frac{I\Delta t}{m_{total}\Delta V} (2)$$

where $I$ is the applied specific current, $\Delta t$ is the discharging time, $m_{total}$ is the total mass of the active material in the two electrodes, and $\Delta V$ is the operating voltage window, which was 2.5 V in the case of our 1M NEt_4_BF_4_ in ACN.

Through GCD findings the energy/power density was obtained through formulas (3) and (4) respectively.

$$E_{d} [Wh kg^{-1}]=\frac{1}{2}\frac{C_{spec;dev.}⸱\Delta V^{2}}{3.6} (3)$$

$$P_{d} [W kg^{-1}]=3600 \frac{E_{d}}{\Delta t} (4)$$

**Supplementary figures**

**Figure S1**. PXRD patterns of MC before and after electrochemical treatment at -2.5 V for 2 h.

**Figure S2.** Representative fitting of the 900-1900 cm^-1^ region of the Raman spectra, using Lorentzian functions of MG-15.

**Figure S3.** Raman combination bands in MG-15, testifying for the turbostratic arrangement of the graphitic layers.


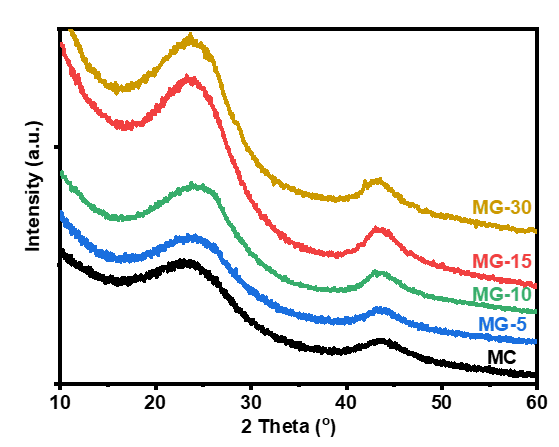


**Figure S4**. PXRDs of MC and MG-T.

**Figure S5.** C1s XPS of MG-5.

**Figure S6.** C1s XPS of MG-30.


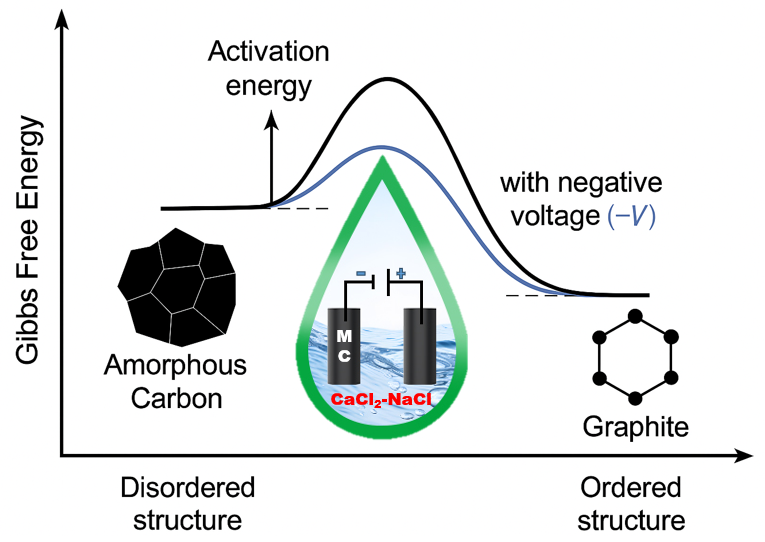


**Figure S7.** The energy diagram illustrates the mechanism by which applying a negative voltage (−V) during heating promotes the transformation of amorphous carbon into graphite.


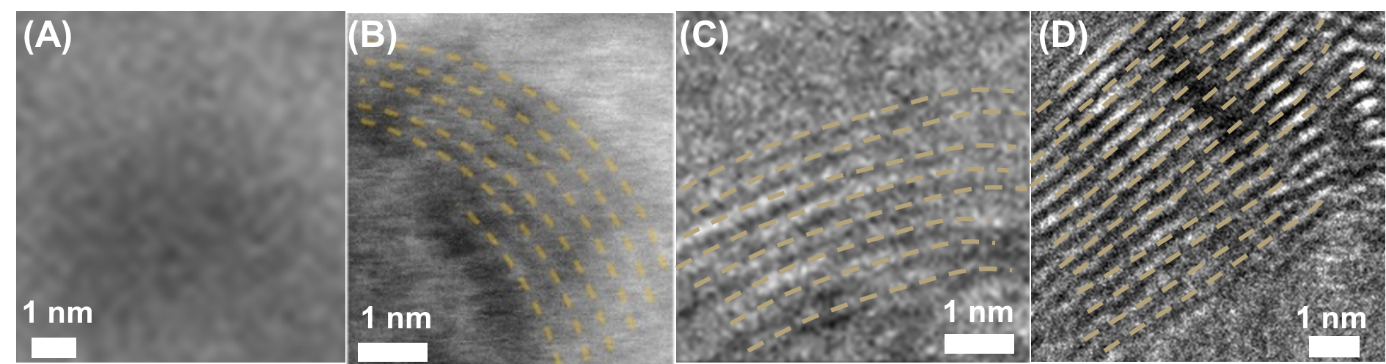


**Figure S8**. TEM images of pristine MC (A), MG-15(B), MG-30(C), MG-60(D).


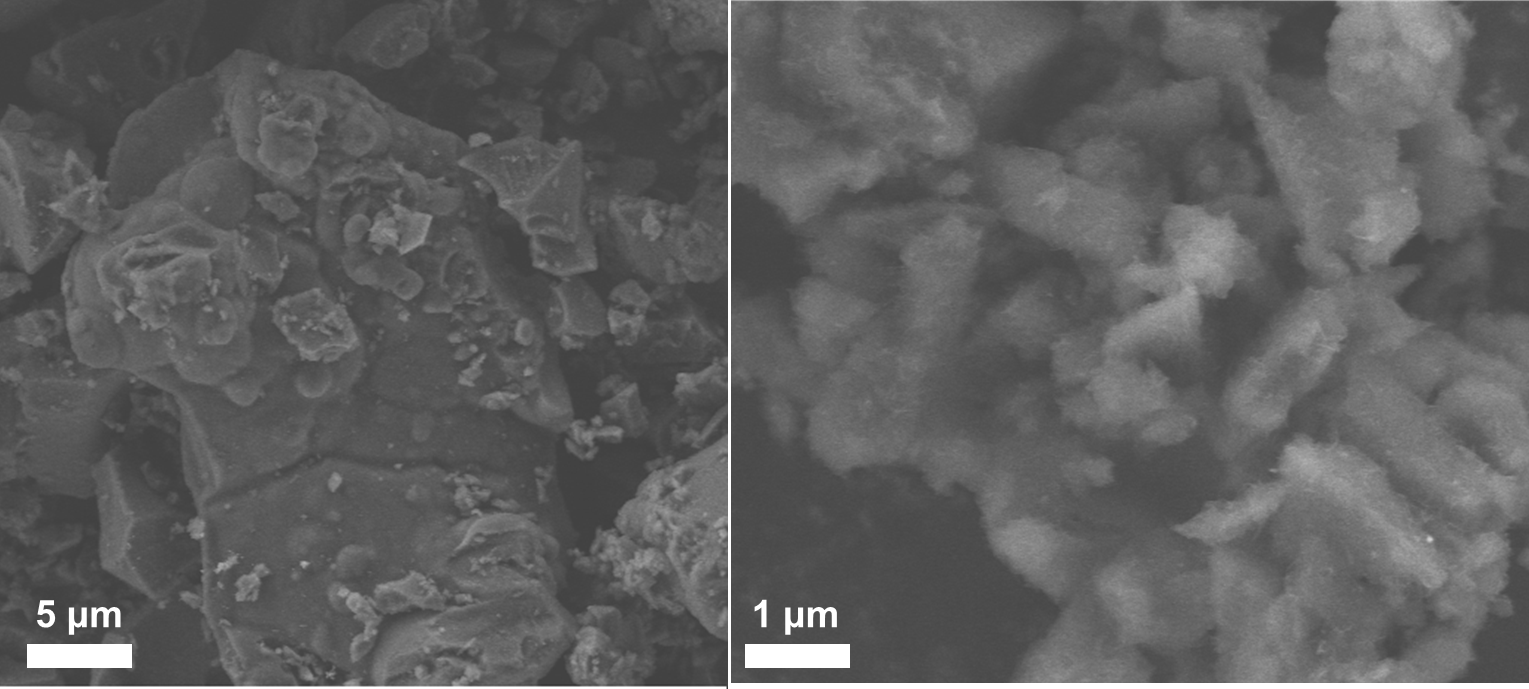


**Figure S9**. SEM images of compressed MG-15.

**Figure S10.** CV curves of MC at different scan rates.

**Figure S11.** CV curves of MG-10 at different scan rates.

**Figure S12.** CV curves of MG-15 at different scan rates.

**Figure S13.** CV curves of MG-30 at different scan rates.

**Figure S14.** The logarithm of the current versus the logarithm of the scan rate, including the fitting line based on CV curves recorded at scan rates of 5, 10, 50, 100, and 200 mV s^−1^

**Figure S15.** Specific capacitance calculated by the integration of CV curves using 1 M H_2_SO_4_ as electrolyte.


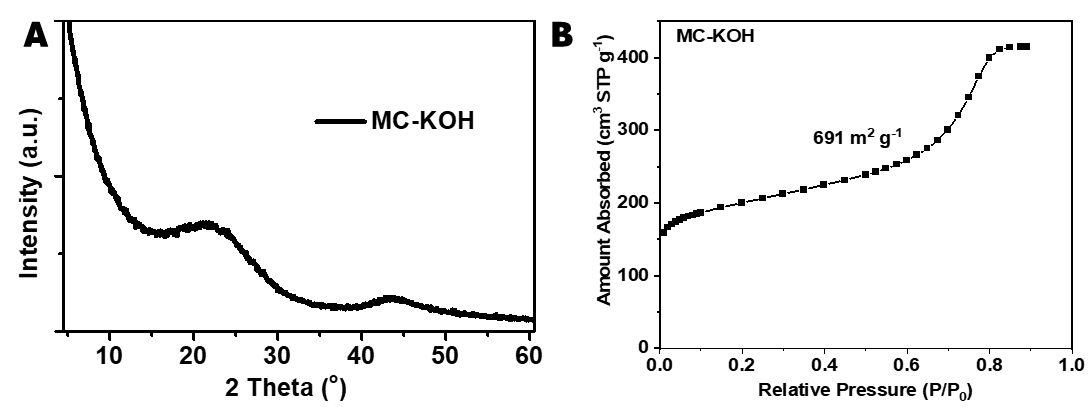


**Figure S16.** Characterization of MC activated by KOH at 700 °C for 2h: (A) PXRD. (B) N_2_ isotherm at 77 K.

**Figure S17**. Voltage holding test using three-electrode configuration at room temperature by applying a constant up-limit potential (0.4 V vs Hg/Hg_2_SO_4_) during aging for up to 400 h. Every 10 h, three GCD cycles were performed between -0.6 V and 0.4 V vs Hg/Hg_2_SO_4_ using a specific current of 5 A g^-1^.

**Figure S18.** CV curves of MC and MG-15 at the scan rate of 10 mV s^-1^ using symmetric cells in 1 M tetraethylammonium tetrafluoroborate (NEt_4_BF_4_) in anhydrous acetonitrile (ACN) electrolyte.

**Figure S19.** Nyquist plot comparison of MC and MG-15

The larger high-frequency semicircle in the Nyquist plot of MG-15 (Figure S17) can be assigned to the enrichment of microporosity.^[2]^ In the case of MG-15, both the total and the micropore surface area increase significantly, with the micropore-to-total specific surface area ratio being 34% higher than that of MC, with the R_ct_ increasing from 11.2 Ohm (MC) to 29.6 Ohm (MG-15). As derived from the intersection of the Nyquist plots with the horizontal axis, the equivalent series resistance (ESR), which stands for the sum of the electrolyte’s ionic resistance, the intrinsic resistance of the active material, and the contact resistance between the active material and the current collector, drops from 2.1 Ohm (MC) to 1.1 Ohm (MG-15), testifying for the improved conductivity of mesoporous graphite.


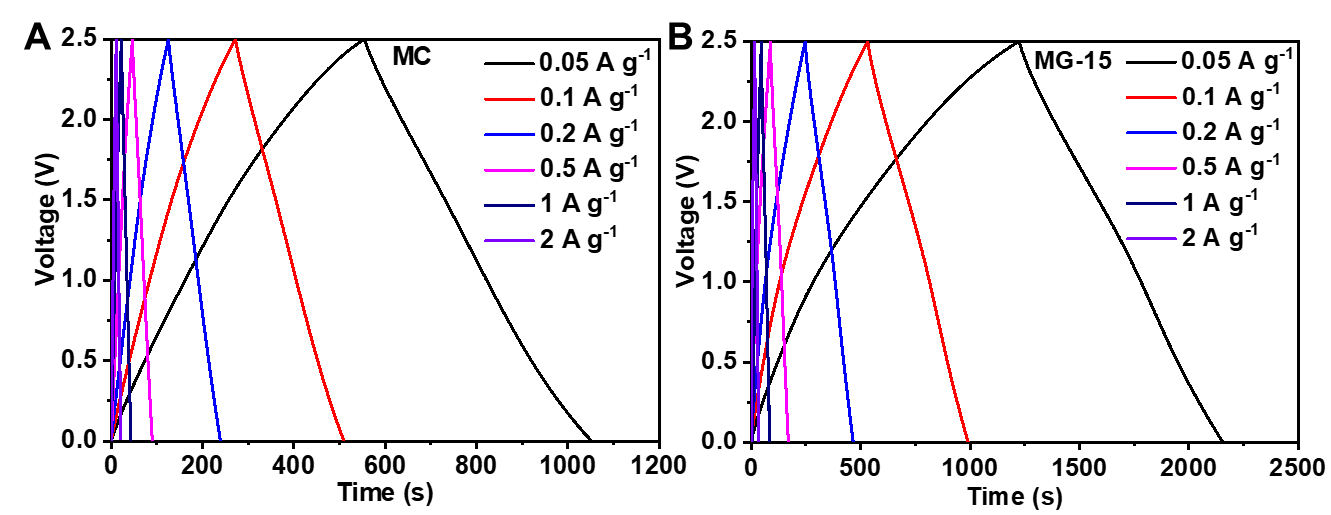


**Figure S20**. GCD curves of symmetric supercapacitors using 1M NEt_4_BF_4_ in ACN electrolyte, using (A) MC and (B) MG-15 electrode materials.

**Figure S21.** Specific capacitance calculated from GCD curves for symmetric supercapacitors using 1M NEt_4_BF_4_ in ACN electrolyte.

**Figure S22.** Ragone plot (energy density vs power density) for symmetric supercapacitors using 1M NEt_4_BF_4_ in ACN electrolyte.

**Table S1** Fitting parameters of combined model with interacting polydisperse sphere and correlation length.

|  | MC | MG-15 | MG-15-(NaCl-CaCl_2_) |
| --- | --- | --- | --- |
| Volume Fraction (scale) | 0.200632 | 0.200848 | 0.119179 |
| mean radius (Å) | 47.07086 | 46.7864 | 74.49596 |
| polydisp (sig/avg) | 0.273898 | 0.300818 | 0.821617 |
| SLD Pore Space (A-2) | 0 | 0 | 4.54E-06 |
| SLD Solid Matrix (A-2) | 3.52E-06 | 3.10E-06 | 3.10E-06 |
| Porod Scale | 1.02E-10 | 1.72E-07 | 8.27E-05 |
| Porod Exponent | 5.041181 | 4.190877 | 3.197554 |
| Lorentzian Scale | 1.145195 | 2.33503 | 0.13979 |
| Lor Screening Length [Å] | 18.16534 | 13.68158 | 8.854755 |
| Lorentzian Exponent | 2.340777 | 2.4638 | 2.89668 |
| Bgd [1/cm] | 0.03425 | 0.043953 | 0.017942 |
| Reduce Chi^2 | 15.44 | 16.78 | 30.27 |

**Table S2**. Summary of the Surface Area, Porosity of MC before and after electrochemical treatment

| Sample | SA_total_ (m^2^ g^−1^)^a^ | SA_micro_ (m^2^ g^−1^)^b^ | V_total_ (cm^3^ g^−1^)^c^ | V_total_ (cm^3^ g^−1^)^c^ |
| --- | --- | --- | --- | --- |
| MC | 397 | 139 | 0.45 | 0.06 |
| MG-5 | 497 | 228 | 0.53 | 0.11 |
| MG-10 | 706 | 350 | 0.61 | 0.16 |
| MG-15 | 867 | 409 | 0.68 | 0.19 |
| MG-30 | 767 | 218 | 0.54 | 0.13 |
| MG-60 | 650 | 125 | 0.59 | 0.07 |
| ^a^Obtained via N_2_ isotherms at 77 K. ^b^Calculated over the relative pressure range P/P0 = 0.2−0.4. ^c^Calculated at the point of P/P_0_ = 0.99 | | | | |

**Reference**

[1] X. Wang, J. S. Lee, C. Tsouris, D. W. DePaoli, S. Dai, *J. Mater. Chem.* **2010**, *20*, 4602-4608.

[2] J. Gamby, P. L. Taberna, P. Simon, J. F. Fauvarque, M. Chesneau, *J. Power Sources* **2001**, *101*, 109-116.
